# Supplementary material for: Genotype and Phenotype Characterization of Rhinolophus sp. Sarbecoviruses from Vietnam: Implications for Coronavirus Emergence
Source: Viruses. 2023 Sep 8;15(9):1897. doi: 10.3390/v15091897 (PMC10536463; doi:10.3390/v15091897)
Supplement: Supplementary file 1 [file viruses-15-01897-s001.zip › Supp data_revised/Supp Figure 1_distance matrix Vietnam only_revised.pdf]

ORF1a		1	2	3	4	5	6	7
1	RtVN21-29	–	95,19	94,59	94,59	99,66	99,66	99,66
2	RtVN21-192	96,21	–	92,43	92,45	95,12	95,12	95,12
3	RtVN21-193	96,81	95,30	–	99,82	94,58	94,58	94,58
4	RsVN21-195	96,85	95,32	99,84	–	94,58	94,58	94,58
5	RtVN21-197	99,50	96,28	96,97	97,01	–	100,00	100,00
6	RtVN21-200	99,50	96,28	96,97	97,01	100,00	–	100,00
7	RtVN21-201	99,50	96,28	96,97	97,01	100,00	100,00	–

NT identity (%)

AA identity (%)

ORF1b		1	2	3	4	5	6	7
1	RtVN21-29	–	94,23	92,54	92,54	96,40	96,39	96,39
2	RtVN21-192	99,24	–	91,70	91,72	97,54	97,54	97,53
3	RtVN21-193	99,09	99,09	–	99,88	92,78	92,77	92,77
4	RsVN21-195	99,05	99,05	99,96	–	92,78	92,78	92,78
5	RtVN21-197	99,32	99,85	99,16	99,13	–	100,00	99,99
6	RtVN21-200	99,32	99,85	99,16	99,13	100,00	–	100,00
7	RtVN21-201	99,32	99,85	99,16	99,13	100,00	100,00	–

NT identity (%)

AA identity (%)

S		1	2	3	4	5	6	7
1	RtVN21-29	–	82,47	79,03	79,03	82,49	82,47	82,47
2	RtVN21-192	86,64	–	78,75	78,80	99,84	99,81	99,81
3	RtVN21-193	83,51	83,60	–	99,95	78,78	78,75	78,75
4	RsVN21-195	83,59	83,68	99,92	–	78,83	78,80	78,80
5	RtVN21-197	86,64	99,92	83,60	83,68	–	99,97	99,97
6	RtVN21-200	86,64	99,92	83,60	83,68	100,00	–	100,00
7	RtVN21-201	86,64	99,92	83,60	83,68	100,00	100,00	–

NT identity (%)

AA identity (%)

ORF3a		1	2	3	4	5	6	7
1	RtVN21-29	–	90,18	81,70	81,70	90,18	90,18	90,06
2	RtVN21-192	90,91	–	90,06	90,06	100,00	100,00	99,88
3	RtVN21-193	82,18	89,45	–	99,76	90,06	90,06	89,94
4	RsVN21-195	81,82	89,09	99,64	–	90,06	90,06	89,94
5	RtVN21-197	90,91	100,00	89,45	89,09	–	100,00	99,88
6	RtVN21-200	90,91	100,00	89,45	89,09	100,00	–	99,88
7	RtVN21-201	90,55	99,64	89,09	88,73	99,64	99,64	–

NT identity (%)

AA identity (%)

E		1	2	3	4	5	6	7
1	RtVN21-29	–	97,40	96,97	96,97	97,40	97,40	97,40
2	RtVN21-192	97,40	–	98,70	98,70	100,00	100,00	100,00
3	RtVN21-193	97,40	100,00	–	100,00	98,70	98,70	98,70
4	RsVN21-195	97,40	100,00	100,00	–	98,70	98,70	98,70
5	RtVN21-197	97,40	100,00	100,00	100,00	–	100,00	100,00
6	RtVN21-200	97,40	100,00	100,00	100,00	100,00	–	100,00
7	RtVN21-201	97,40	100,00	100,00	100,00	100,00	100,00	–

NT identity (%)

AA identity (%)

M		1	2	3	4	5	6	7
1	RtVN21-29	–	95,35	94,59	94,74	95,35	95,35	95,35
2	RtVN21-192	99,55	–	95,05	95,20	100,00	100,00	100,00
3	RtVN21-193	97,75	97,30	–	99,85	95,05	95,05	95,05
4	RsVN21-195	98,20	97,75	99,55	–	95,20	95,20	95,20
5	RtVN21-197	99,55	100,00	97,30	97,75	–	100,00	100,00
6	RtVN21-200	99,55	100,00	97,30	97,75	100,00	–	100,00
7	RtVN21-201	99,55	100,00	97,30	97,75	100,00	100,00	–

NT identity (%)

AA identity (%)

ORF6		1	2	3	4	5	6	7
1	RtVN21-29	–	97,88	89,06	89,06	97,35	97,35	97,35
2	RtVN21-192	98,41	–	90,62	90,62	99,47	99,47	99,47
3	RtVN21-193	85,94	87,50	–	100,00	90,10	90,10	90,10
4	RsVN21-195	85,94	87,50	100,00	–	90,10	90,10	90,10
5	RtVN21-197	96,83	98,41	85,94	85,94	–	100,00	100,00
6	RtVN21-200	96,83	98,41	85,94	85,94	100,00	–	100,00
7	RtVN21-201	96,83	98,41	85,94	85,94	100,00	100,00	–

NT identity (%)

AA identity (%)

ORF7a		1	2	3	4	5	6	7
1	RtVN21-29	–	97,56	91,87	91,87	97,83	97,83	97,83
2	RtVN21-192	98,37	–	91,33	91,33	99,19	99,19	99,19
3	RtVN21-193	94,31	94,31	–	100,00	91,60	91,60	91,60
4	RsVN21-195	94,31	94,31	100,00	–	91,60	91,60	91,60
5	RtVN21-197	99,19	99,19	95,12	95,12	–	100,00	100,00
6	RtVN21-200	99,19	99,19	95,12	95,12	100,00	–	100,00
7	RtVN21-201	99,19	99,19	95,12	95,12	100,00	100,00	–

NT identity (%)

AA identity (%)

ORF7b		1	2	3	4	5	6	7
1	RtVN21-29	–	99,26	94,81	94,81	99,26	99,26	99,26
2	RtVN21-192	100,00	–	94,81	94,81	100,00	100,00	100,00
3	RtVN21-193	97,78	97,78	–	100,00	94,81	94,81	94,81
4	RsVN21-195	97,78	97,78	100,00	–	94,81	94,81	94,81
5	RtVN21-197	100,00	100,00	97,78	97,78	–	100,00	100,00
6	RtVN21-200	100,00	100,00	97,78	97,78	100,00	–	100,00
7	RtVN21-201	100,00	100,00	97,78	97,78	100,00	100,00	–

NT identity (%)

AA identity (%)

ORF8		1	2	3	4	5	6	7
1	RtVN21-29	–	86,07	79,78	79,78	96,45	96,45	96,45
2	RtVN21-192	86,89	–	74,04	74,04	89,62	89,62	89,62
3	RtVN21-193	78,69	73,77	–	100,00	79,51	79,51	79,51
4	RsVN21-195	78,69	73,77	100,00	–	79,51	79,51	79,51
5	RtVN21-197	97,54	89,34	80,33	80,33	–	100,00	100,00
6	RtVN21-200	97,54	89,34	80,33	80,33	100,00	–	100,00
7	RtVN21-201	97,54	89,34	80,33	80,33	100,00	100,00	–

NT identity (%)

AA identity (%)

N		1	2	3	4	5	6	7
1	RtVN21-29	–	97,95	95,51	95,51	97,63	97,63	97,63
2	RtVN21-192	99,05	–	96,06	96,06	98,82	98,82	98,82
3	RtVN21-193	97,16	97,16	–	99,92	96,38	96,38	96,38
4	RsVN21-195	97,16	97,16	100,00	–	96,38	96,38	96,38
5	RtVN21-197	98,82	98,82	97,16	97,16	–	100,00	100,00
6	RtVN21-200	98,82	98,82	97,16	97,16	100,00	–	100,00
7	RtVN21-201	98,82	98,82	97,16	97,16	100,00	100,00	–

NT identity (%)

AA identity (%)

ORF10		1	2	3	4	5	6	7
1	RtVN21-29	–	100,00	100,00	100,00	100,00	100,00	100,00
2	RtVN21-192	100,00	–	100,00	100,00	100,00	100,00	100,00
3	RtVN21-193	100,00	100,00	–	100,00	100,00	100,00	100,00
4	RsVN21-195	100,00	100,00	100,00	–	100,00	100,00	100,00
5	RtVN21-197	100,00	100,00	100,00	100,00	–	100,00	100,00
6	RtVN21-200	100,00	100,00	100,00	100,00	100,00	–	100,00
7	RtVN21-201	100,00	100,00	100,00	100,00	100,00	100,00	–

NT identity (%)

AA identity (%)
