# Supplementary material for: Genotype and Phenotype Characterization of Rhinolophus sp. Sarbecoviruses from Vietnam: Implications for Coronavirus Emergence
Source: Viruses. 2023 Sep 8;15(9):1897. doi: 10.3390/v15091897 (PMC10536463; doi:10.3390/v15091897)
Supplement: Supplementary file 1 [file viruses-15-01897-s001.zip › Supp data_revised/Supp Figure 5_phylo nt all sarbeco RBD_revised.pdf]

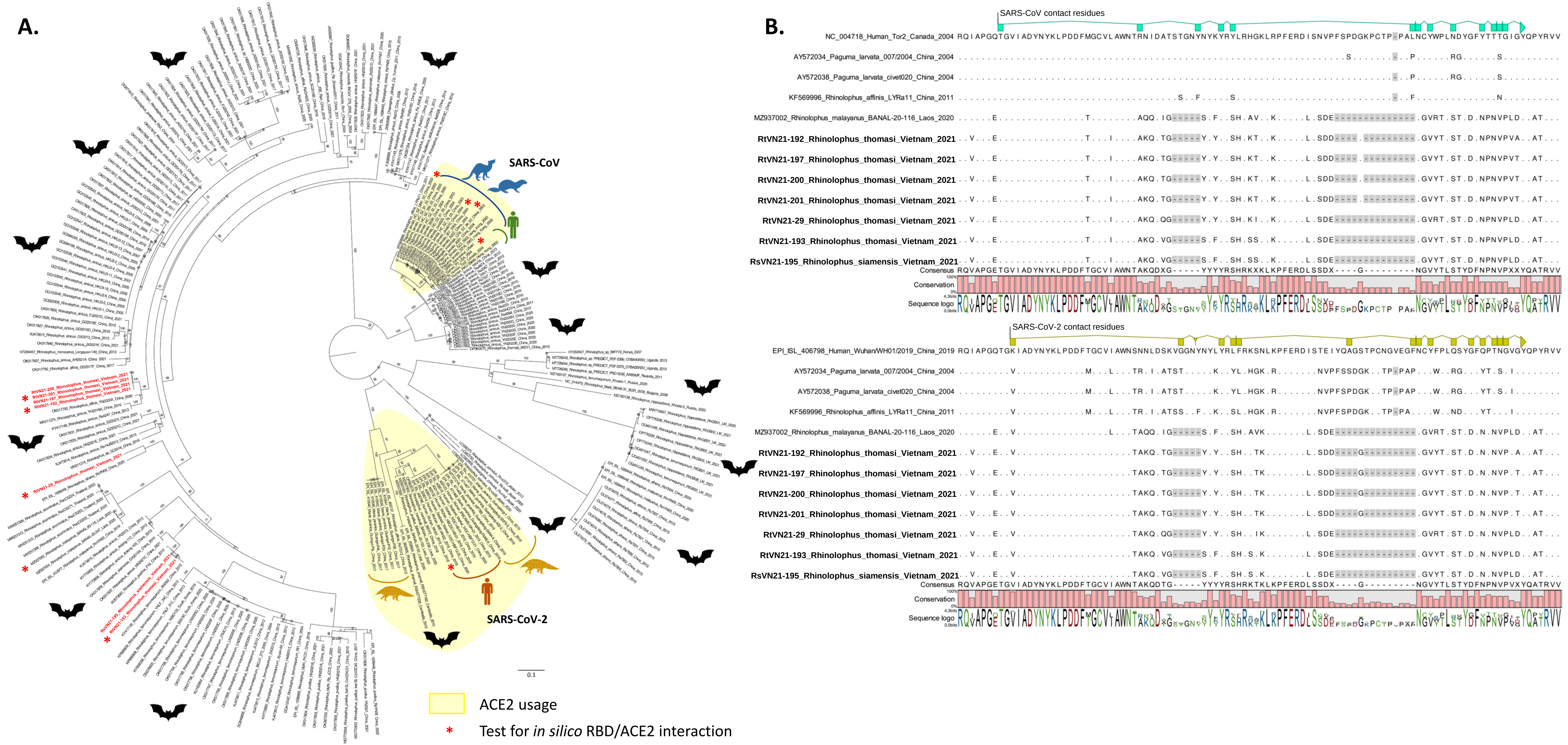

A.
SARS-CoV
SARS-CoV-2
RtVN21-200_Rhinolophus_thomasi_Vietnam_2021
RtVN21-201_Rhinolophus_thomasi_Vietnam_2021
RtVN21-197_Rhinolophus_thomasi_Vietnam_2021
RtVN21-192_Rhinolophus_thomasi_Vietnam_2021
RtVN21-29_Rhinolophus_thomasi_Vietnam_2021
RsVN21-195_Rhinolophus_siamensis_Vietnam_2021
RtVN21-193_Rhinolophus_thomasi_Vietnam_2021
0.1
ACE2 usage
* Test for *in silico* RBD/ACE2 interaction
B.
SARS-CoV contact residues
NC_004718_Human_Tor2_Canada_2004
AY572034_Paguma_larvata_007/2004_China_2004
AY572038_Paguma_larvata_civet020_China_2004
KF569996_Rhinolophus_affinis_LYRa11_China_2011
MZ937002_Rhinolophus_malayanus_BANAL-20-116_Laos_2020
RtVN21-192_Rhinolophus_thomasi_Vietnam_2021
RtVN21-197_Rhinolophus_thomasi_Vietnam_2021
RtVN21-200_Rhinolophus_thomasi_Vietnam_2021
RtVN21-201_Rhinolophus_thomasi_Vietnam_2021
RtVN21-29_Rhinolophus_thomasi_Vietnam_2021
RtVN21-193_Rhinolophus_thomasi_Vietnam_2021
RsVN21-195_Rhinolophus_siamensis_Vietnam_2021
Consensus
Conservation
Sequence logo
SARS-CoV-2 contact residues
EPI_ISL_406798_Human_Wuhan/WH01/2019_China_2019
AY572034_Paguma_larvata_007/2004_China_2004
AY572038_Paguma_larvata_civet020_China_2004
KF569996_Rhinolophus_affinis_LYRa11_China_2011
MZ937002_Rhinolophus_malayanus_BANAL-20-116_Laos_2020
RtVN21-192_Rhinolophus_thomasi_Vietnam_2021
RtVN21-197_Rhinolophus_thomasi_Vietnam_2021
RtVN21-200_Rhinolophus_thomasi_Vietnam_2021
RtVN21-201_Rhinolophus_thomasi_Vietnam_2021
RtVN21-29_Rhinolophus_thomasi_Vietnam_2021
RtVN21-193_Rhinolophus_thomasi_Vietnam_2021
RsVN21-195_Rhinolophus_siamensis_Vietnam_2021
Consensus
Conservation
Sequence logo
