# Supplementary figures and images for: Genotype and Phenotype Characterization of Rhinolophus sp. Sarbecoviruses from Vietnam: Implications for Coronavirus Emergence
Source: Viruses. 2023 Sep 8;15(9):1897. doi: 10.3390/v15091897 (PMC10536463; doi:10.3390/v15091897)

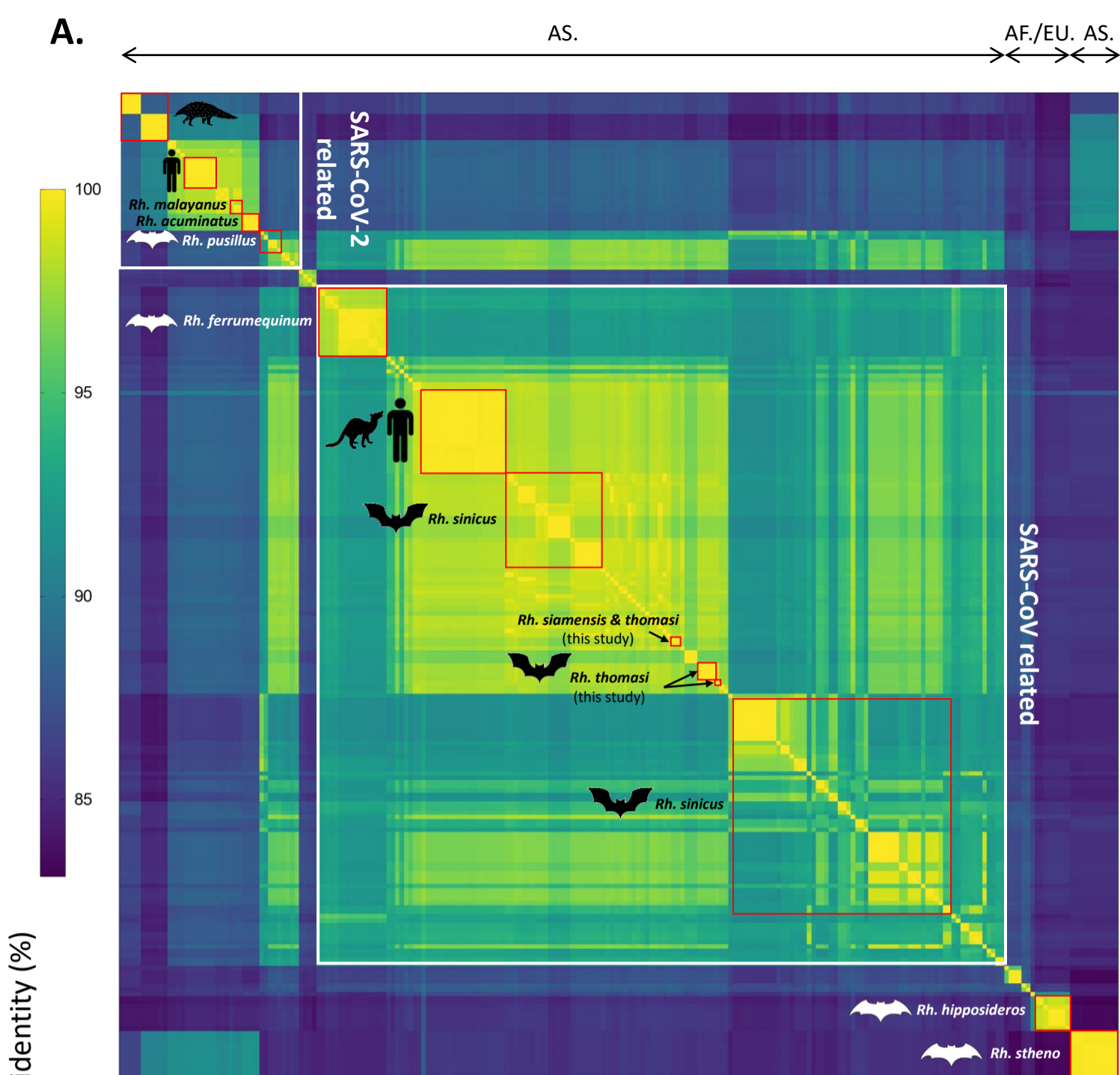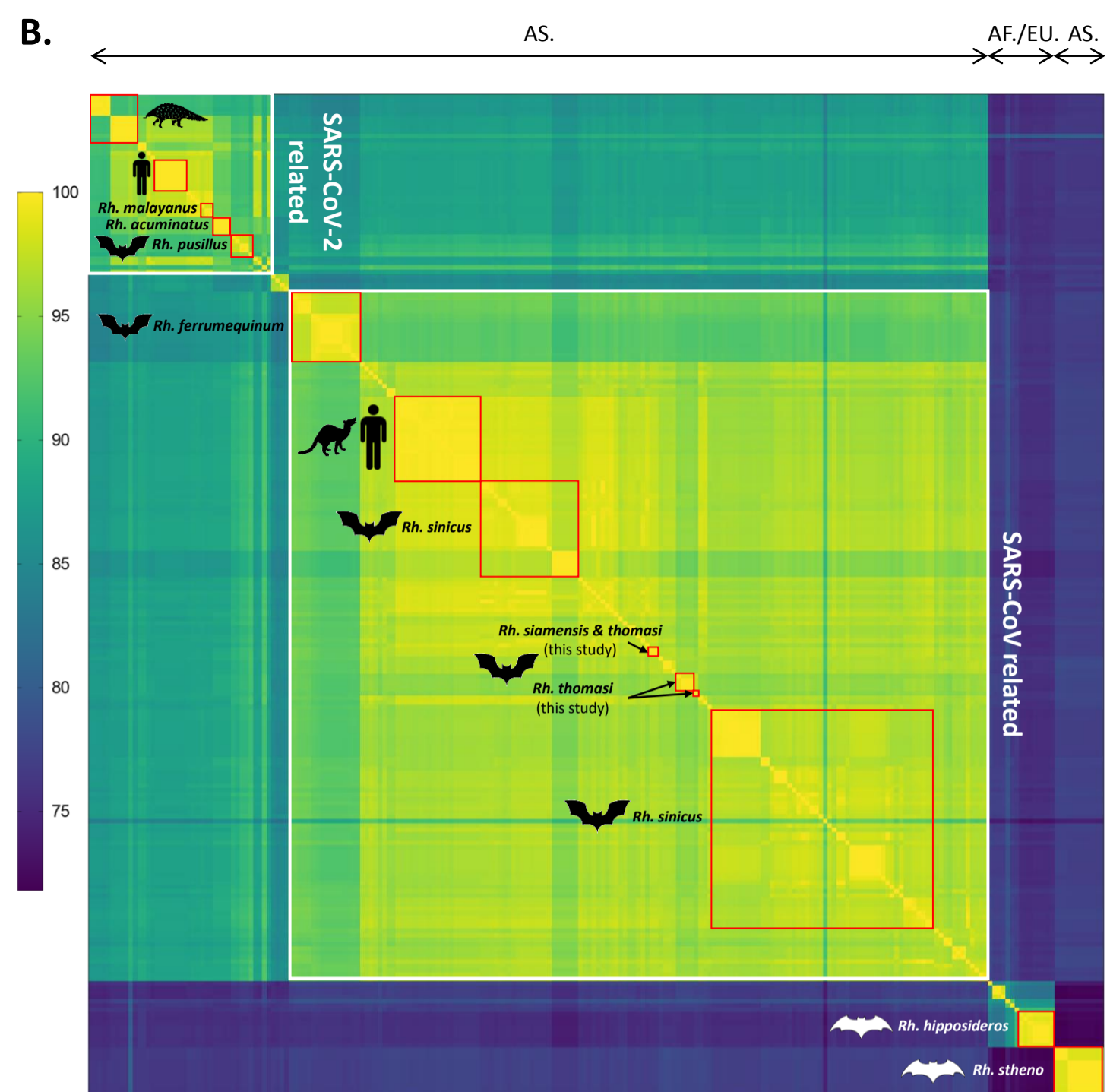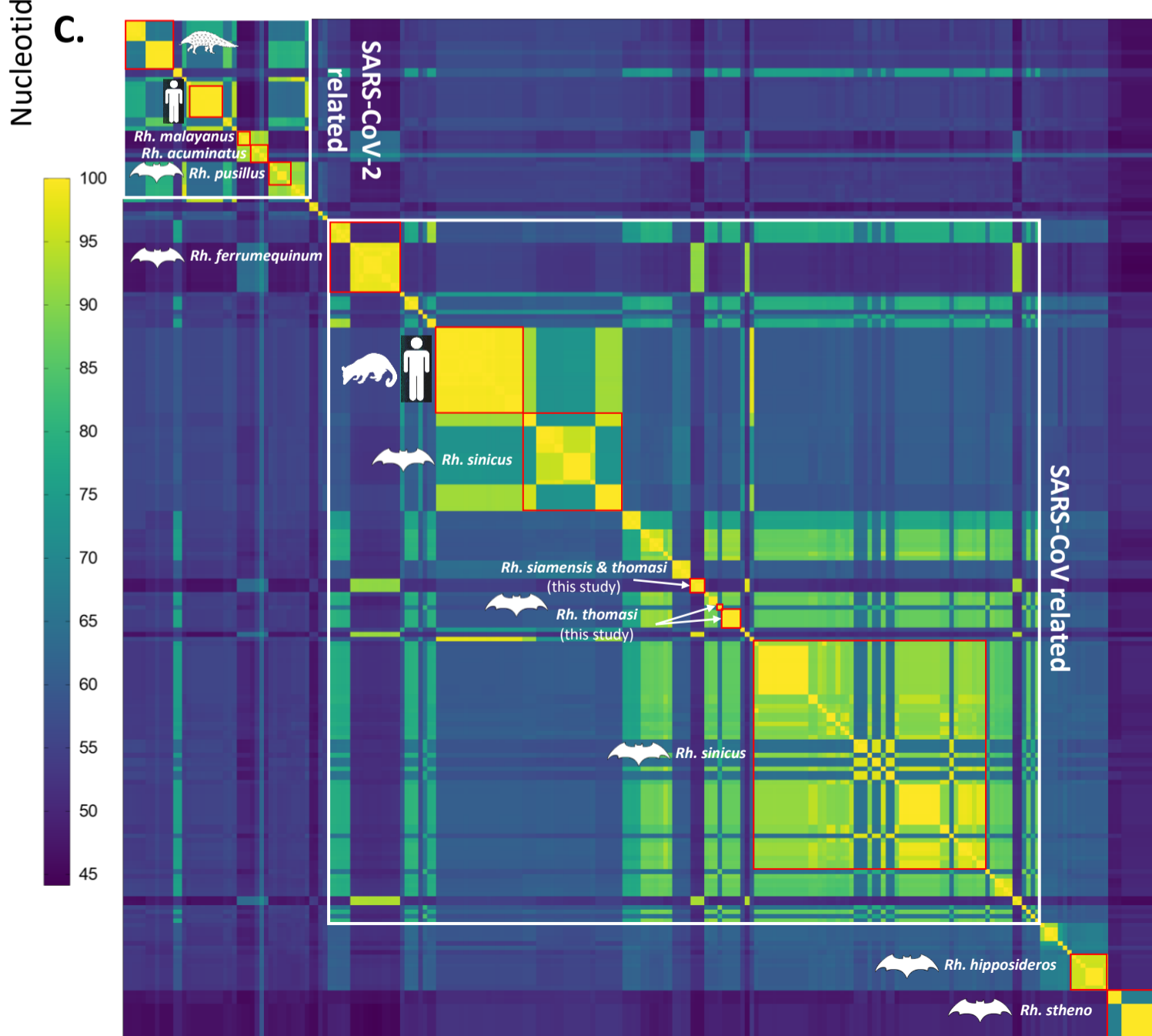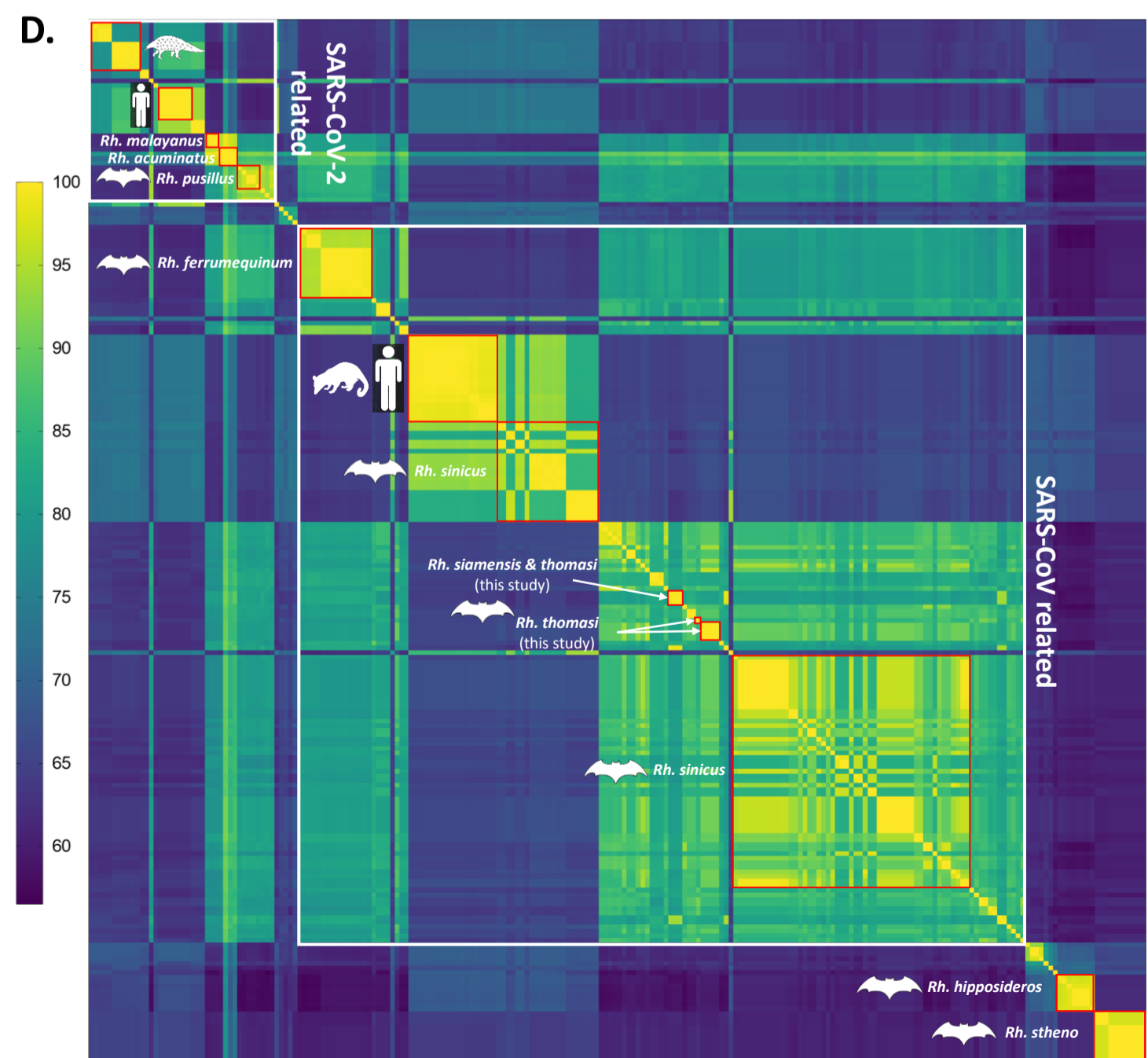

Supplement: Supplementary file 1 [file viruses-15-01897-s001.zip › Supp data_revised/Supp Figure 2_heatmap RdRP_N_S-NTD_S-RBD all sarbeco nt_revised.pdf]

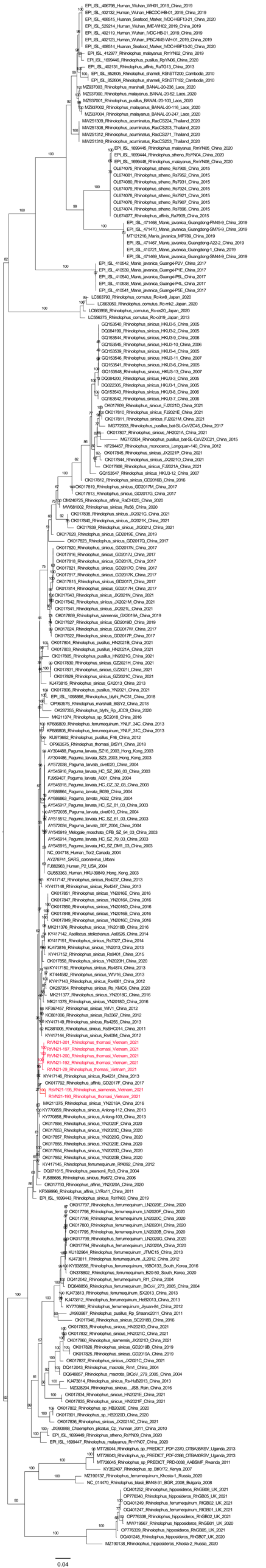

0.04

Supplement: Supplementary file 1 [file viruses-15-01897-s001.zip › Supp data_revised/Supp Figure 3_ALN A2C-C2A all sarbeco complete genome nt focus RdRP.pdf]

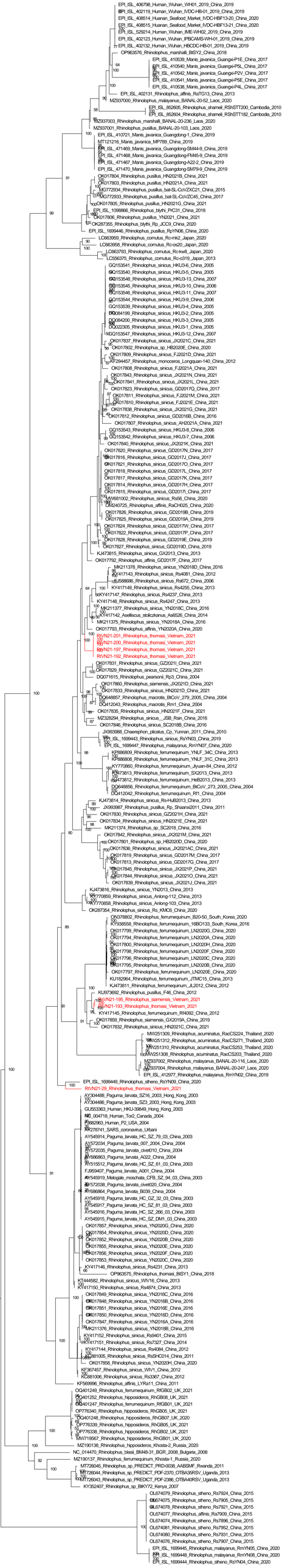

Supplement: Supplementary file 1 [file viruses-15-01897-s001.zip › Supp data_revised/Supp Figure 4_ALN A2C-C2A all sarbeco complete genome nt focus Spike.pdf]
